# Characterization of Gonadal Transcriptomes from Nile Tilapia (*Oreochromis niloticus*) Reveals Differentially Expressed Genes

**DOI:** 10.1371/journal.pone.0063604

**Published:** 2013-05-03

**Authors:** Wenjing Tao, Jing Yuan, Linyan Zhou, Lina Sun, Yunlv Sun, Shijie Yang, Minghui Li, Sheng Zeng, Baofeng Huang, Deshou Wang

**Affiliations:** Key Laboratory of Freshwater Fish Reproduction and Development (Ministry of Education), Key Laboratory of Aquatic Science of Chongqing, School of Life Science, Southwest University, Chongqing, P.R. China; Temasek Life Sciences Laboratory, Singapore

## Abstract

Four pairs of XX and XY gonads from Nile tilapia were sequenced at four developmental stages, 5, 30, 90, and 180 days after hatching (dah) using Illumina Hiseq^TM^ technology. This produced 28 Gb sequences, which were mapped to 21,334 genes. Of these, 259 genes were found to be specifically expressed in XY gonads, and 69 were found to be specific to XX gonads. Totally, 187 XX- and 1,358 XY-enhanced genes were identified, and 2,978 genes were found to be co-expressed in XX and XY gonads. Almost all steroidogenic enzymes, including *cyp19a1a*, were up-regulated in XX gonads at 5 dah; but in XY gonads these enzymes, including *cyp11b2*, were significantly up-regulated at 90 dah, indicating that, at a time critical to sex determination, the XX fish produced estrogen and the XY fish did not produce androgens. The most pronounced expression of steroidogenic enzyme genes was observed at 30 and 90 dah for XX and XY gonads, corresponding to the initiation of germ cell meiosis in the female and male gonads, respectively. Both estrogen and androgen receptors were found to be expressed in XX gonads, but only estrogen receptors were expressed in XY gonads at 5 dah. This could explain why exogenous steroid treatment induced XX and XY sex reversal. The XX-enhanced expression of *cyp19a1a* and *cyp19a1b* at all stages suggests an important role for estrogen in female sex determination and maintenance of phenotypic sex. This work is the largest collection of gonadal transcriptome data in tilapia and lays the foundation for future studies into the molecular mechanisms of sex determination and maintenance of phenotypic sex in non-model teleosts.

## Introduction

Sex determination and maintenance involve complex processes with many interacting events. Genetic females and males undergo different selective pressures, some of which operate in opposite directions, to produce differences in gene expression and phenotypic divergence [1]. Differences in gene expression between the two sexes, which can be spectacular, have been the subject of intense scrutiny. A few genes have been identified as candidate master sex determining genes. These include *SRY/Sry* in eutherian mammals [2], *Dmrt1* in chickens [3], *dmy* in medaka [4], *Dmw* in the African clawed frog [5], *amhy* in the Patagonian pejerrey [6], and *irf9y* in the rainbow trout [7] (see Table S1 for the symbols and full names of all genes mentioned in the text). In addition to those candidate sex-determining genes, a number of relatively conserved genes have been shown to be involved in sex determination. These include *sox9* [8], *wt1* [9], *foxl2* [10], *dax1* [11], and *sf1* [12]. The joint action of proteins encoded by these genes leads to the developmental and physiological divergence between XX and XY gonads. However, the molecular basis for sex determination and maintenance has not been well characterized. For this reason, the assessment of gene expression profiles at the transcriptomic level during gonadal development is essential to understanding sex determination and maintenance of phenotypic sex.

Microarray and EST analyses have long been used to elucidate the molecular mechanisms underlying sex determination and differentiation and to identify genes differentially expressed between the two sexes in mice [13], [14], [15], rats [16], Mozambique tilapia [17], zebrafish [18], and rainbow trout [19], [20]. Recent studies have shown that RNA-Seq is more suitable to quantify transcripts expressed at low levels than microarrays or EST analysis [21], [22], [23]. This is because RNA-seq verifies direct transcript profiling without compromise or bias. For this reason, it has been used in transcriptome profiling studies in relation to sex determination and differentiation of several organisms, including snakes [24], clams [1], platyfish [25], and rainbow trout [7]. However, no studies performed on non-model vertebrates to date have described either XX or XY gonad transcriptomes at different developmental stages or any comprehensive gene expression profiles in relation to specific events, such as sex determination, gametogenesis, or steroidogenesis (such as estrogen biosynthesis).

Estrogens play a pivotal role in sex differentiation in non-eutherian vertebrates such as rainbow trout [26], tilapia [27], catfish [28], chicken [29], and tammar wallaby [30]. The P450 aromatases, encoded by *cyp19a1a* and *cyp19a1b*, are steroidogenic enzymes responsible for the synthesis of estrogens from androgens in fish. One recent report places estrogens and *cyp19a1a* in pivotal positions that regulate not only ovarian but also testicular differentiation in both gonochoristic and hermaphrodite fish species [31]. DNA methylation of *cyp19a1a*, for example, has been reported to be involved in temperature-dependent shifts in the sex ratios of European seabass [32]. The Nile tilapia (*Oreochromis niloticus*) is a gonochoristic fish in which sex determination is governed by the XX-XY system. It is currently the fish species with the second highest production worldwide behind carps [33]. As the growth rate of Nile tilapia males during the grow-out period is substantially higher than that of females [34], breeding and culturing all-male populations of the Nile tilapia is a promising way of increasing the yield. Investigation of the molecular mechanisms of sex determination and maintenance is of great significance in aquaculture. In this species, the regions governing sex determination have been reported to be LG1 [35], [36], LG3 [37], [38], [39], and LG23 [40], [41] by different researchers using different strains and families. This suggests that various strains might have different sex chromosomes. It is well documented that environmental temperature also affects the phenotypic sex of tilapia [42], [43]. Recent studies from our group demonstrated that *sf1*, *foxl2* [44], and *dmrt1* [45] are involved in the transcriptional regulation of *cyp19a1a*. Although some previous studies have provided expression profiles of several key steroidogenic enzyme genes in early developmental stages of tilapia [46], [47], [48], [49], the exact roles played by steroids at genomic and transcriptomic levels during tilapia sex determination and differentiation are still unknown. For this reason, further identification of the expression profiles of genes involved in gonadal development using RNA-Seq may help to elucidate the gene regulatory network governing sex determination and subsequent maintenance of phenotypic sex. In this study, we sequenced the transcriptomes of four pairs of XX and XY gonads at four developmental stages. Our aim was to identify differences in gene expression profiles between the two sexes and among different developmental stages that can then be used to elucidate the molecular and genetic mechanisms of sex determination and maintenance of phenotypic sex. These data may provide a very useful genomic resource for future studies of male and female gonadal development and for the selection of candidate genes involved in these processes in Nile tilapia. These data also provide the basis for future comparison of different downstream effects of gene expression induced by LG1Y, LG3Y, and LG23Y sex determination genes in different strains.

## Materials and Methods

### Fish

The fish used in the present study were provided by Professor Nagahama's laboratory of the National Institute for Basic Biology, Japan. The fish were pure *Oreochromis niloticus*, which has a XY mode of sex determination. Sex-specific DNA markers (unpublished data) provided evidence that the sex determining region of the genome was located on LG23. Tilapia were reared in re-circulating aerated freshwater tanks at 26°C prior to use. All-XX and all-XY progenies were produced by crossing the pseudo-male (XX, sperm-producing) and super-male (YY) with the normal female (XX). XX and XY fish were dissected to produce testicular and ovarian samples for RNA isolation at four critical stages of tilapia gonadal development: the critical time for sex determination and differentiation (5 days after hatching [dah]; 300 gonads pooled for each sex), initiation of germ cell meiosis and oogenesis in XX gonads (30 dah, 150 gonads pooled for each sex), initiation of germ cell meiosis or spermatogenesis in XY gonads (90 dah, 3 gonads pooled for each sex), and sperm maturation and vitellogenesis in XY and XX gonads (180 dah, 3 gonads pooled for each sex).

All experiments were conducted in accordance with the regulations provided by the Guide for Care and Use of Laboratory Animals and were approved by the Committee of Laboratory Animal Experimentation at Southwest University.

### RNA isolation

**Figure 1:**
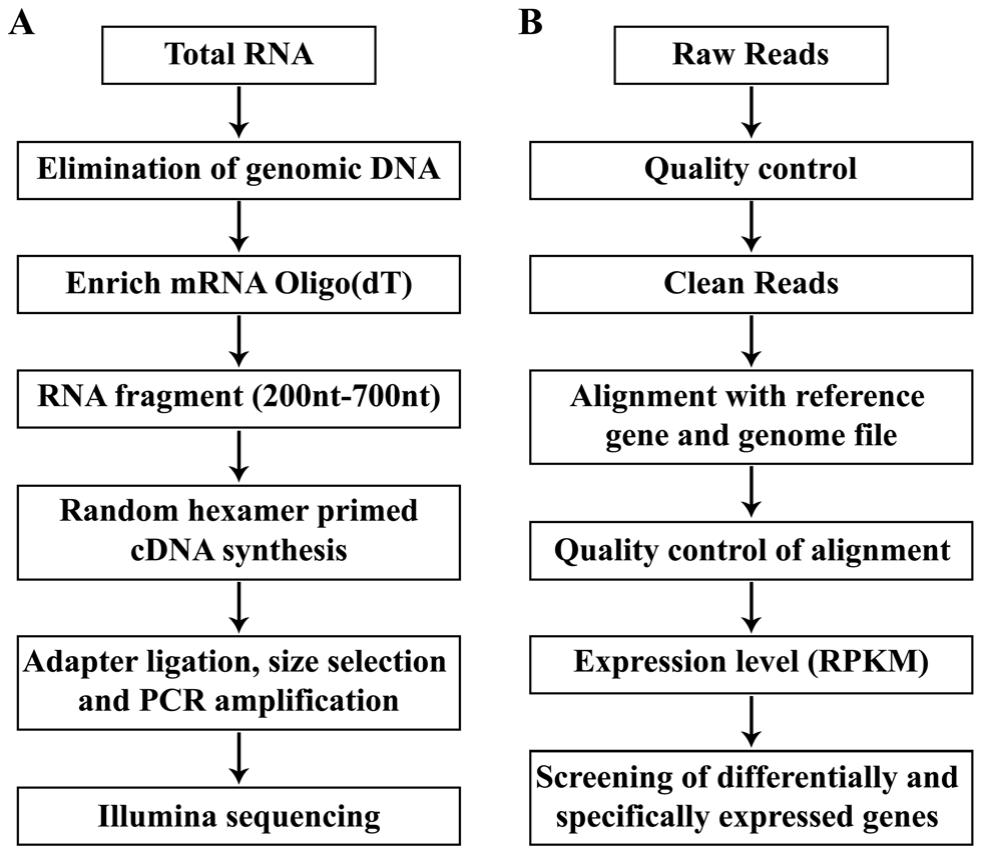
Pipeline of experiment (A) and bioinformatics analysis (B).

Total RNA was extracted from each sample using Trizol Reagent (Invitrogen, Carlsbad, CA, U.S.) according to the manufacturer's instructions. The extracted RNA was further treated with DNase1 (RNase-free 5 U/μL) to eliminate genomic DNA contamination. The concentration and integrity of RNA were determined using a NanoDrop spectrophotometer (NanoDrop Technologies, Wilmington, DE, U.S.) and Agilent 2100 Bioanalyzer (Agilent Technologies, Palo Alto, CA, U.S.). The RNA with OD260/280 = 1.8–2.1, OD260/230≥1.7, c≥150 ng/µL, RNA integrity number (RIN)≥7.0 and 28S/18S>1.0 were performed for mRNA enrichment using oligo (dT) beads. The enriched mRNA was disrupted into short fragments (200–700 nt) using fragmentation buffer. These short fragments were used as templates, first-strand cDNA was synthesized using an Invitrogen cDNA synthesis kit (Invitrogen, Carlsbad, CA, U.S.), followed by synthesis of second-strand cDNA using custom second-strand synthesis buffer, dNTPs, RNase H, and DNA polymerase I. A QiaQuick PCR purification kit (Qiagen GmbH, Hilden, Germany) was used to purify these fragments and EB buffer was used for end repair and addition of the poly (A) tail. Then these short fragments were ligated with sequencing adapters. After agarose gel electrophoresis, fragments between 320 and 370 nt were cut from the gel for PCR amplification. In total, eight cDNA libraries were constructed from the eight respective samples. Flow chart A in Figure 1 shows the major steps of the experiment.

### Illumina sequencing, gene read mapping, and calculation of gene expression levels

Sequence reads (2*100 bp pair-end sequencing) pools were generated on an Illumina HiSeq^TM^ 2000 instrument from the Beijing Genomics Institute at Shenzhen (BGI Shenzhen, China) from the eight cDNA libraries. The clean reads from the eight transcriptomes were obtained from raw data by filtering out adaptor-only reads and low-quality reads (reads with Q-value≤20).

The reference genome and gene data from *O. niloticus* (Orenil1.0) were downloaded from the Ensembl web site (http://www.ensembl.org/Oreochromis_niloticus/Info/Index). Clean reads from each library were aligned to the reference genome using TopHat v2.0.6 [50]. This program allows multiple alignments per read (up to 20 by default) and a maximum of 2 mismatches when mapping the reads to the reference genome [51]. The reference genome used lacks both Y loci because the sequenced individual was from an XX clonal line. Because of this, no analysis of any Y-locus specific alleles that may have diverged in sequence beyond the TopHat alignment filter settings could be performed. Because the mapped read count of a given gene is influenced by its length and sequencing discrepancy, it cannot be used to represent the gene expression level properly. The reads per kb per million reads (RPKM) method was used to calculate gene expression levels [21]. The formula is as follows:
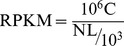
Here, C and L indicate the mapped read count and length of a given gene, respectively, and N indicates total mapped read count of a given library. In this way, the RPKM can be directly used to compare the differences in gene expression among genes from the same sample and from different samples (see Fig. 1B for the major steps of bioinformatics analysis).

### Identification of sex-specifically expressed, differentially expressed, and co-expressed genes

Audic's method was used to identify differentially expressed genes between two libraries [52]. The threshold for the *P*-value was determined using false discovery rate (FDR), and was widely set at 10^−2^ [53], [54]. In this study, “FDR≤10^−2^” and “|log_2_ (XX_RPKM/XY_RPKM)|≥1” were used to identify differentially expressed genes (DIGs) and specifically expressed genes (SEGs). Genes from each stage were divided into two categories: i) genes expressed in both XX and XY gonads; and ii) genes expressed specifically either in the XX or XY gonad only. Among the genes expressed in both XX and XY gonads, those meeting both “FDR≤10^−2^” and “|log_2_ (XX_RPKM/XY_RPKM)|≥1” statistical criteria were classified as DIGs, whereas all the remaining ones were called XX and XY co-expressed genes (COGs). Among the genes expressed specifically either in the XX or XY gonad, those meeting both “FDR≤10^−2^” and “|log_2_ (XX_RPKM/XY_RPKM)|≥1” statistical criteria were grouped as XX/XY-SEGs, whereas the rest were designated as ND-SEGs. This way, all genes were classified into five types: COGs, XX-DIGs, XY-DIGs, XX/XY-SEGs, and ND-SEGs (Table S2).

### Validation of transcriptomic data

Validation of the expression profiles of the selected DIGs was carried out in two ways: 1) for characterized genes, transcriptome data were compared to published data; and 2) for uncharacterized genes without published data, real-time PCR (qPCR) was performed as described previously to verify the expression profile obtained from the transcriptome data. To perform qPCR, gonads were dissected from XX and XY tilapia at 5, 30, 90, or 180 dah, and total RNA was isolated from each sample and reverse-transcribed using MMLV reverse transcriptase (Invitrogen, Carlsbad, CA, U.S.) according to the manufacturer's protocol. An online real-time PCR primer design tool, GenScript Primer Design (http://www.genscript.com/cgi-bin/tools/primer_genscript.cgi), was used to design the primers (listed in Table S3). The SYBR Green I Master Mix (TaKaRa, Dalian, China) was used for qPCR. qPCR products were quantified using an Applied Biosystems Prism 7500-fast real-time PCR system. The PCR reactions were initiated by denaturation at 95°C for 5 min; followed by 40 amplification cycles at 95°C for 15 s and 60°C for 30 s. Dissociation protocols were used to measure melting curves and to control non-specific signals from the primers. Four β-actin genes were found in Nile tilapia. One of them (GenBank ID: XM_003443127) has been proved to be uniformly expressed in XX and XY gonads. It has been widely used as an internal control for qPCR [55], [56], [57]. It was also used as an internal control in the present study. Primers for β-actin were according to Yoshiura *et al.* [55]. Relative expression levels were calculated as described previously [56]. The statistical package GraphPad Prism (GraphPad Software, Inc.) was used to analyze data from all experiments. The averages of the relative quantities of biological replications (3–4) were used in a two-tailed Student's t-test with a 95% confidence level (*P*<0.05) to determine the significance with respect to differences between gene expression values for ovary *vs.* testis.

### Data availability

Short-read data of the eight sequenced transcriptomes were deposited in NCBI's Short Read Archive at http://www.ncbi.nlm.nih.gov/sra/under the accession number SRA055700.

## Results

### RNA-seq and read mapping

Sequencing of the eight cDNA libraries derived from XX and XY gonads at 5, 30, 90, and 180 dah generated 361 million reads, encompassing 28 Gb of sequences. Each stage was represented by approximately 45 million reads, a tag density sufficient for the quantitative analysis of gene expression profiles. Of all the clean reads from the eight libraries, 76.12% were matched to genomic locations. The remaining 23.88% were not matched. In total 21,334 genes were found to be expressed in tilapia gonads, providing abundant data for the analysis of tilapia gonadal development. The statistics for individual transcriptomes are shown in Table 1.

**Table 1:** Read counts, gene counts, and RPKM values of developing tilapia XX/XY gonads.

Stage	Read count	Gene count	Number of genes with RPKM≥1	Number of genes with RPKM≥5
5 dah XX	53,333,332	17,224	8,028	3,313
5 dahXY	51,111,110	18,022	11,670	5,637
30 dah XX	53,140,336	20,537	15,783	10,971
30 dahXY	52,579,466	20,696	16,553	12,205
90 dah XX	24,763,572	18,203	13,163	10,001
90 dahXY	24,872,242	19,825	16,640	12,315
180 dahXX	51,485,734	19,341	13,745	10,489
180 dahXY	50,258,478	20,659	16,146	11,936
Total	361,544,270	21,334	19,652	16,201

### Validation of transcriptomic data

Previous studies have described XX-enhanced expression of *foxl2* [46], *fgf16*, and *fgf20b* [57] and XY-enhanced expression of *dmrt1* [46] during tilapia gonadal development. In the present work, all genes displayed consistent expression profiles in transcriptome data (Table S4). Comparison of the transcriptome data with the qPCR results from four selected DIGs (*foxh1*, *foxj1a*, *42sp50*, and *eef1a1b*) revealed similar expression profiles, even though the exact fold-change varied (Table 2).

**Table 2:** Validation of transcriptome results by qPCR.

	*foxh1*		*foxj1a*		*42sp50*		*eef1A1b*	
	RPKM	qPCR	RPKM	qPCR	RPKM	qPCR	RPKM	qPCR
5 dah XX	0.00	ND	0.67	ND	0.18	ND	3.78	ND
5 dah XY	0.28	ND	2.90	ND	0.62	ND	8.34	ND
30 dah XX	1.27	1.00	1.38	1.00	10.99	1.00	59.64	1.00
30 dah XY	0.76	0.44	1.18	0.79	13.36	0.54	23.55	0.48
90 dah XX	66.32	3.64	0.07	0.12	1127.70	38.45	5.92	0.11
90 dah XY	2.92	0.38	81.15	9.77	26.33	3.15	497.17	11.32
180 dah XX	70.19	8.26	0.02	0.28	363.13	24.34	0.67	0.30
180 dah XY	2.86	1.99	103.15	13.39	14.83	3.59	475.29	32.00

Note: “ND” indicates not determined. Transcriptome data are shown as RPKM and qPCR results are shown as fold changes relative to 30 dah XX.

### Global gene expression in tilapia gonads

**Figure 2:**
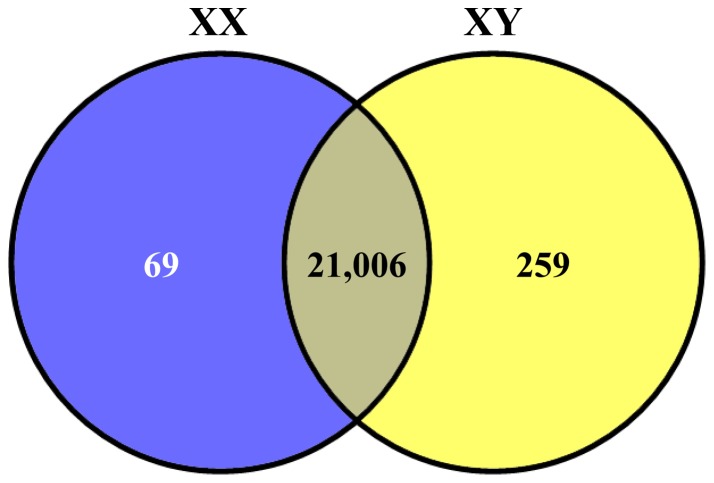
Venn diagram showing the number of genes expressed in XX (21,075) and XY (21,265) gonads throughout at the four stages of development. A total of 21,006 genes were expressed in both XX and XY gonads, but 69 and 259 genes were specifically expressed in XX and XY gonads, respectively.

Of the 21,334 genes from eight transcriptomes, 21,006 were found to be co-expressed in both XX and XY gonads, and 69 and 259 genes were found to be expressed exclusively in XX and XY gonads, respectively (Figure 2). The number of XY-SEGs from all four developmental stages was nearly four-fold greater than for XX-SEGs.

**Figure 3:**
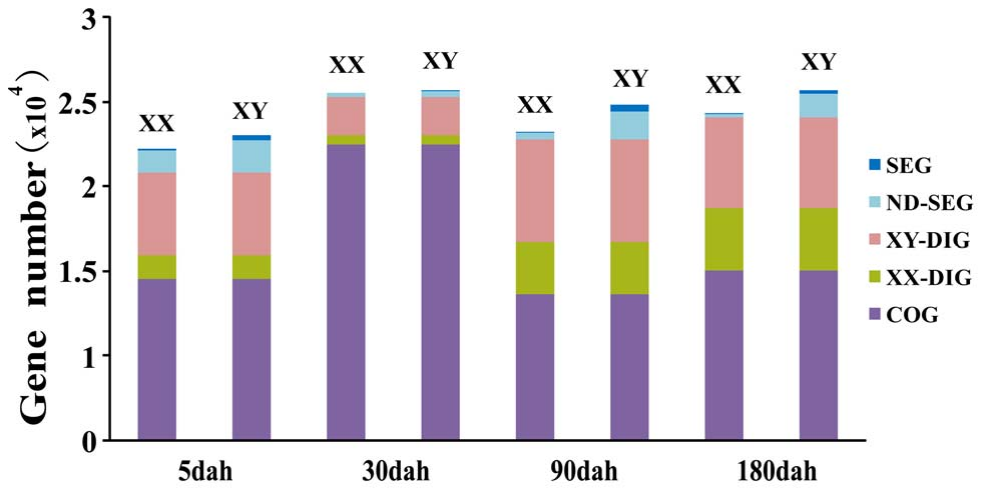
Five types of genes in XX and XY transcriptomes of tilapia gonads. A hierarchical clustering of differentially expressed transcripts (FDR≤10^−2^ and |log_2_ (XX_RPKM/XY_RPKM)|≥1) generated five types of genes. “SEGs” indicate genes specific to either XX or XY. “ND-SEGs” indicate SEGs that did not meet statistical criteria. “XX-DIGs” indicate XX differentially expressed genes. “XY-DIGs” indicate XY differentially expressed genes. “COGs” indicate XX and XY co-expressed genes.

**Figure 4:**
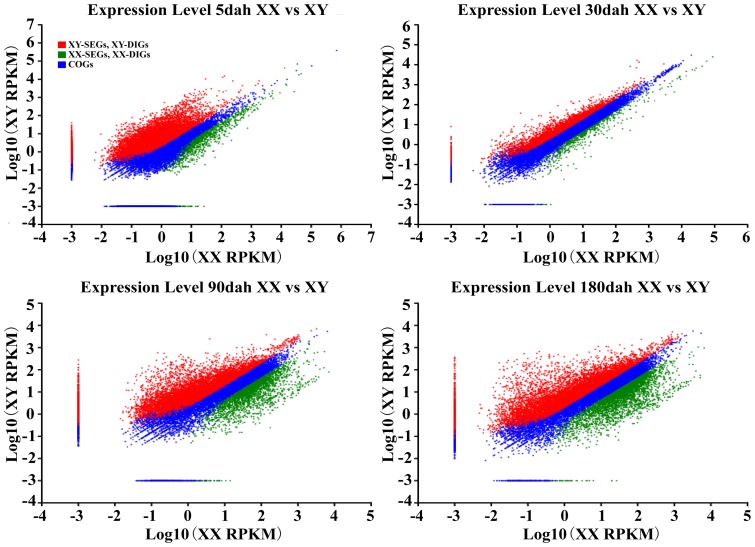
Scatter plots showing gene expression profiles in XX and XY tilapia gonads at four developmental stages (FDR≤10^−2^ and |log_2_ (XX_RPKM/XY_RPKM)|≥1). More genes were found to be up-regulated in XY samples than in XX samples at all four developmental stages and the difference in expression between the two sexes at 30 dah was the least significant of any point in time measured here.

The global analysis of the number of genes for each transcriptome showed that more genes were expressed in the XY than in the XX gonads at every developmental stage examined (Figure 3). There were 9,549, 17,492, 8,652, and 10,030 COGs expressed in gonads at 5, 30, 90, and 180 dah, respectively; 30 dah constituted the largest change in magnitude across the four developmental stages. There were 91, 0, 37, and 10 XX-SEGs and 296, 3, 392, and 187 XY-SEGs at 5, 30, 90, and 180 dah, respectively. The number of SEGs was negatively correlated with the number of COGs expressed at all four stages. While the numbers of XX-DIGs at 5, 30, 90, and 180 dah were 1,394, 512, 3,057, and 3,653, respectively, the corresponding numbers for XY DIGs were: 4,882, 2,254, 6,065, and 5,412, respectively. Overall, more SEGs and DIGs were observed in XY gonads. A simple comparison of the scatter plots of the gene expression profiles at each developmental stage also revealed that there were more up-regulated genes in XY (red plots) than in XX (green plots) at all four developmental stages (Figure 4), which is consistent with the enhanced SEGs and DIGs observed in XY gonads (Figure 3). Among the four developmental stages, the most substantial differences between the two sexes were observed at 180 dah, followed by 90, 5, and 30 dah in decreasing order (Figure 3 and Figure 4). When the gene expression profiles of the same sex at different stages were compared, the 90 and 180 dah groups were found to share the most similarity in expression profile (Figure S1).

### Co-expressed genes (COGs) in XX and XY gonads

Despite the significant differences in gene expression observed between XX and XY gonad transcriptomes, a preliminary analysis revealed that the COGs expressed at every developmental stage made up a signficant proportion of the total (Figure 3). Among these COGs, 2,978 were expressed at all four developmental stages; 8,017 were co-expressed at 5 and 30 dah; 7,597 were co-expressed at 30 and 90 dah; and 6,768 were co-expressed at 90 and 180 dah (Figure S2). We analyzed COGs with high RPKM values at every developmental stage and found robust co-expression of ribosomal protein genes. Ribosomal protein genes *rpl3*, *rpl5*, and *rpl12* were found to be highly expressed at all four different developmental stages. Detailed information with respect to COGs (gene name and RPKM) throughout all four developmental stages is shown in Table S5. Genes critical to gonadal development in both sexes, including *lamc2* [58], *gpc4* [59], *igfbp3* [60], and *ddx46* [61], are also shown in Table S5.

### Identification of XX- and XY-enhanced genes

SEGs and DIGs expressed at 5 dah and in at least two other developmental stages in XX and XY gonads were considered XX- and XY-enhanced genes, respectively. Based on these criteria, 187 and 1,358 genes were identified as XX- and XY-enhanced genes (Figure S3 and Figure S4). In general, all XX- and XY-enhanced genes could be divided into three groups: 1) un-annotated; 2) annotated with known function in sex determination and differentiation; and 3) annotated but without known function in sex determination and differentiation.

Of the 187 XX-enhanced genes, 11 were up-regulated in XX samples at all four developmental stages (Figure S3 and Table S6). The most well-documented XX gonad markers (such as *cyp19a1a*, *cyp19a1b*, and *foxl2*, which are involved in estrogen synthesis and ovarian differentiation), were among the genes here identified as XX-enhanced. Other such genes are essential for female germ-line maintenance and oogenesis. These included *snip1* [62], *wnt5a* [63], *par-3* [64], and *pen* [65]. Among the 1,358 XY-enhanced genes, 225 were up-regulated at all four developmental stages (Figure S4 and Table S7). As expected, *dmrt1* was preferentially expressed in the testis, and the remaining XY-enhanced genes included genes known to be critical to testicular differentiation and spermatogenesis, such as *nanos2* [66], *arhgap42* [67], *dazap1* [68], *hsp70* [69], [70], and *m33* [71].

### Sex steroids and sex steroid receptors

**Figure 5:**
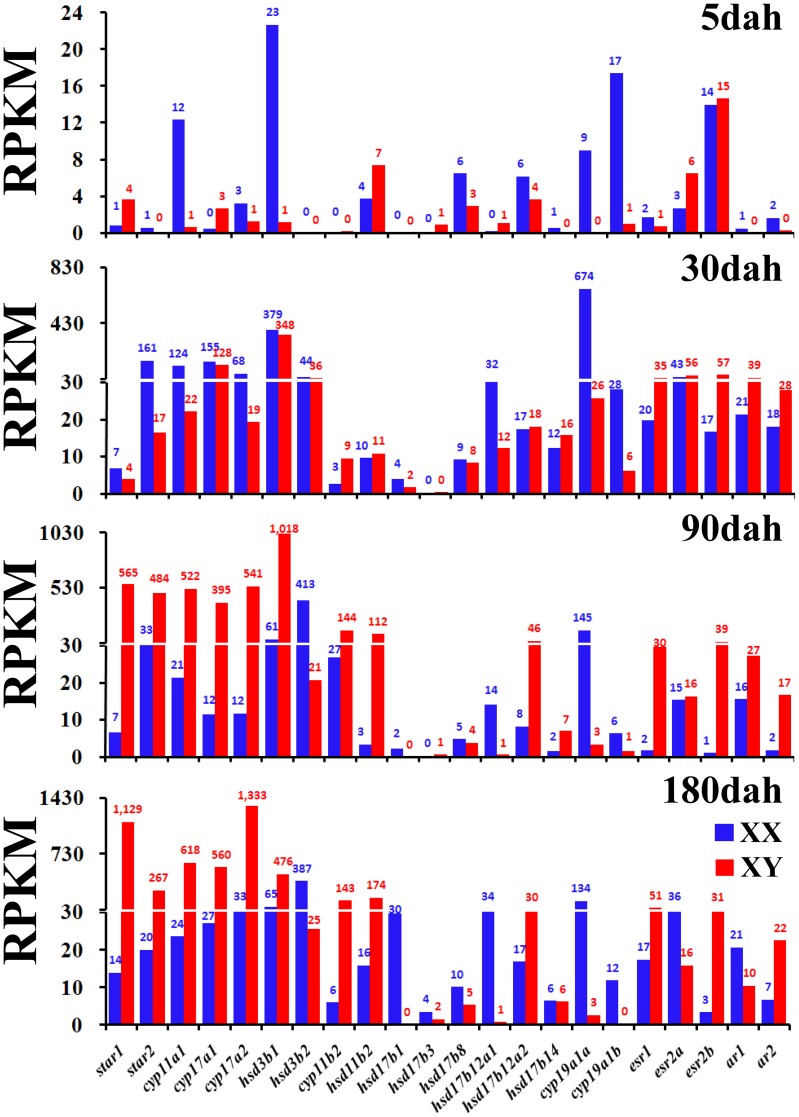
The levels of expression of 17 steroidogenic enzyme genes, three estrogen receptor genes (*esr1*, *esr2a*, and *esr2b*) and two androgen receptor genes (*ar1* and *ar2*) in XX and XY gonads of tilapia at 5, 30, 90, and 180 dah. Numbers within the figure indicate RPKM values.

Genes involved in sex steroid synthesis mostly showed higher expression levels in XX gonads than in XY gonads at 5 dah. The exceptions included *star1*, *cyp17a1*, *hsd11b2*, *esr2a*, and *esr2b*. The total and average RPKM of the listed 17 steroidogenic enzyme genes were 84 and 5 for XX, and 27 and 2 for XY gonads at 5 dah, respectively (Table 3). We observed higher expression levels of steroidogenic enzyme genes such as *star2*, *cyp11a1*, *cyp17a2*, *hsd3b1*, *hsd17b8*, *hsd17b12a2*, *hsd17b14*, *cyp19a1a*, and *cyp19a1b* in XX than in XY gonads at 5 dah (Figure 5). However, *cyp11b2* (encoding the key enzyme 11β-hydroxylase for androgen production in tilapia) was not detected in either XX or XY gonads at 5 dah. From 5 to 30 dah, expression of steroidogenic enzyme genes in both XX and XY gonads underwent an exponential increase, with the total and average reaching 1,728 and 102 for XX and 683 and 40 for XY, respectively. C*yp19a1a* was the most dramatically differentially expressed gene among these 17 genes between 5 and 30 dah. However, the expression profiles of these genes were completely reversed at 90 and 180 dah. During this period, almost all genes were up-regulated in the XY gonads and down-regulated in the XX gonads; with the total and average reaching 3,866 and 227 at 90 dah and 4,772 and 281 at 180 dah for XY gonads, respectively. These numbers diminished to 771 and 45 at 90 dah, and 837 and 49 at 180 dah for XX gonads, respectively. *Hsd17b1* was the most dramatically differentially expressed gene among these 17 steroidogenic enzyme genes at 90 and 180 dah. RPKMs of *star1*, *star2*, *cyp11a1*, *cyp17a1*, *cyp17a2*, and *hsd3b1* were higher than 200, and several exceeded 1,000, in XY gonads, but only a few estrogen-producing enzyme genes, such as *hsd3b2* and *cyp19a1a*, displayed moderate expression in XX gonads. At these two stages, expression of *cyp19a1b* was down regulated, with RPKMs less than 15 even in XX gonads. Unexpectedly, *hsd17bs* exhibited low levels of expression (with RPKM no more than 30) in both XX and XY gonads at all four developmental stages. However, *hsd17b12a1* showed a relatively high level of expression at 30 and 180 dah in XX gonads, and *hsd17b12a2* showed a relatively high level of expression at 90 dah in XY gonads.

**Table 3:** The expression profile of 17 genes* coding for steroidogenic enzymes.

	5 dah		30 dah		90 dah		180 dah	
	XX	XY	XX	XY	XX	XY	XX	XY
Sum**	84	27	1728	683	771	3866	837	4772
Average	5	2	102	40	45	227	49	281
Mostdiff	*cyp19a1a*		*cyp19a1a*		*hsd17b1*		*hsd17b1*	

Note: * For the complete list of genes see Figure 5. ** “Sum” indicates the total RPKM of the 17 steroidogenic enzymes; “average” indicates the average RPKM of the 17 steroidogenic enzymes; “mostdiff” indicates the most dramatically differentially expressed gene among the 17 steroidogenic enzymes at each stage.

At 5 dah, androgen receptors *ar1* and *ar2* were not detectable in XY gonads, but both were detectable in XX gonads. The three estrogen receptors *esr1*, *esr2a*, and *esr2b* were detected in both XX and XY gonads at 5 dah. *Esr2b* was expressed at higher levels in XY gonads at all four developmental stages than in XX gonads. Expression levels of both estrogen and androgen receptors peaked at 30 dah in XY gonads.

## Discussion

### Comprehensive gonadal transcriptome data in vertebrates

Tightly controlled sequential changes in gene expression are required during sex determination and differentiation. Almost all previous surveys of transcriptional regulation of sex determination were conducted using microarray and SAGE [13], [14], [15], [16], [18]. More recent studies on gonadal transcriptomes using RNA-Seq have been conducted. For the most part, these used either the ovary or testis but not both with RNA sampled from a variety of tissues at a variety of developmental stages [25], [72], [73]. Few studies have focused on the gene expression profiles of early undifferentiated gonads. This is because monosex fish are only available for a few species, including tilapia. This is the first study to explore global gene expression profiles of XX and XY gonads at different developmental stages. There are two requirements for obtaining gonadal RNA from tilapia at 5 and 30 dah, the availability of all-XX and all-XY progeny and precise dissection skills. There are currently no comprehensive sequences or comparative transcriptomic analyses of ovarian and testicular development in vertebrates that were performed using RNA-seq. In this way, the 21,075 and 21,265 genes expressed in the ovary and testis reported herein represent a large and previously unavailable transcriptomic resource for gonads in tilapia. Of all the clean reads, about 23.88% were not mapped to any specific sequence. These unmapped reads can be grouped into five categories: 1) reads located in the uncovered area of the reference genome; 2) reads whose sequencing errors failed to meet TopHat alignment filter settings; 3) short reads located at the border of alternatively spliced exons; 4) reads from contaminated genomes, especially from microbes, and 5) reads whose sequences differed from those of the strain or individual used for genome sequencing.

### Co-expressed genes (COGs) in ovaries and testes

Even though XX and XY gonads display significant morphologic differences, they also share some similarities. For example, both are composed of germ cells and somatic cells that rely on protein synthsis for proper functionality. In the present study, the expression of 2,978 COGs showed with no significant differences between the two sexes at any point in the four developmental stages examined. These COGs included the following: 1) *lamc2*, which is a glycoprotein, constitutes major non-collagenous basement membranes in mice [58]; 2) *gpc4*, whose complex and developmentally regulated expression pattern suggest potential involvement in one or more signaling pathways in several different developing tissues in mice [59]; 3) *igfbp3*, which has been proven to possess important physiological functions in overall development and reproduction in the common carp [60]; and 4) *ddx46*, a member of the DEAD-box family that is highly expressed in both XX and XY gonads at all stages examined. This class of proteins participates in several aspects of RNA metabolism and translational events, including pre-mRNA splicing, ribosomal biogenesis, nucleo-cytoplasmic transport, protein translation, and RNA degeneration, which ultimately orient organelle genes toward specific biological functions [61]. Gene expression in germ cells also requires DEAD-box family members for the temporal uncoupling of transcription and translation.

### Sex differences in gene expression profiles in gonads

The identification and characterization of the XX- and XY-enhanced genes at different developmental stages is of vital importance to our understanding how these molecular differences regulate XX and XY gonadal differentiation and development. In this study, we analyzed the gene expression profiles of the ovary and testis at 5, 30, 90, and 180 dah and identified 187 XX- and 1,358 XY-enhanced genes. Some of the XX-enhanced genes were found to be involved in XX gonad development. These included the following: 1) *snip1*, which has been reported to be required for oocyte development in mice [62]; 2) *wnt5a*, which has been characterized as important to the female reproductive system in mice [63]; 3) *par-3*, which serves as a platform for assembly of proteins involved in development of egg polarity in mice [64]; and 4) *pen*, which encodes an importin-α2 subfamily member and helps in the completion of primordial follicle formation and the newborn ovary homeobox in zebrafish [65]. Some of the XY-enhanced genes have been proven to be various XY gonadal marker genes. These include the following: 1) *nanos2*, which is predominantly expressed in male germ cells, without which mice cannot produce spermatogonia [66]; 2) *arhgap42*, which mediates the regulation of the cytoskeletal network and junctional dynamics of modified testis-specific cell-cell actin-based adherens junctions in humans [67]; 3) *dazap1*, which is highly expressed in human and mouse testes and plays a key role in mRNA transport during spermatogenesis [68]; 4) *hsp70*, which has been identified as potentially important to the male testis and germ cells of rabbits [69], [70]; and 5) *m33*, the lack of which may cause male-to-female sex reversal in mice [71].

When different developmental stages were compared with respect to sex, gene expression profiles were found to be more similar at 90 and 180 dah for both XX and XY gonads. There were more genes with similar levels of expression in XX gonads than in XY gonads, indicating similar functions in oogenesis and spermatogenesis.

Some of the XX- and XY-enhanced genes we identified are known to show sexual dimorphism in their expression in the ovary or testis of several other species illustrating the reliability of the selection standard in our analyses. Comparision of our transcriptomic data with qPCR results and previous reports further validates the selection strategy used for identification of XX- and XY-enhanced genes in ovary and testis. Our analyses also identified many previously uncharacterized XX- and XY-enhanced genes. From this point of view, transcriptomic analysis is indeed a useful approach to the identification of other sex-specific and sex-enhanced genes. Further functional characterization of these genes using transgenic overexpression, knockout strategies, and knockdown strategies may help elucidate the molecular mechanisms controlling sex determination and gonadal development in teleosts.

### Number of genes expressed in XY gonads and XX gonads (SEGs and DIGs)

In the present study, more genes were found to be specifically expressed (SEGs) in XY gonads than in XX gonads. This difference was detectable at any of the four developmental stages analyzed and in total as well (259 XY-SEGs *vs*. 69 XX-SEGs). In addition, more enhanced genes (1,358 *vs.* 187) were found to be expressed in XY gonads than in XX gonads. When each stage was analyzed separately, more DIGs and SEGs were also found to be expressed in XY gonads than in XX gonads. The XX gonad is larger in size and more active in steroidogenesis during early developmental stages than the XY gonad (5 dah). However, only 91 genes were found to be expressed specifically in XX gonads, and 296 genes were expressed specifically in XY gonads. We speculate that early expression of a sex-determining gene in XY gonads may trigger an early male-specific gene expression pattern before any female pattern emerges. This pattern may involve the XY-SEGs. The exact roles of these XY-SEGs in tilapia sex determination, differentiation, and maintenance of phenotypic sex are still a mystery. One of the possible roles of these XY-SEGs may be to antagonize the genes responsible for the ovarian pathway or promote the genes involved in testicular development. In this way, functional characterization of these genes and re-examination of the current paradigm is needed to elucidate more fully the molecular basis for sex determination and differentiation in fish and in other vertebrates.

### Importance of estrogen and androgen to sex determination and maintenance of sex phenotype

Sex reversal is one of the most powerful approaches used to study the roles of estrogen and androgens in sex determination and phenotypic maintenance. Fadrozole (an aromatase inhibitor) and 17*β*-estradiol (E2) have been widely used to induce XX and XY sex reversal, respectively, in tilapia [74], [75]. Typically, when treatment is administered during the period covering 3–6 dah, phenotypic sex can be successfully reversed in both sexes. It is also well documented that the sex-specific expression of *foxl2* and *cyp19a1a* in XX gonads and *dmrt1* in XY gonads during early gonadal differentiation (5–6 dah) is critical to differentiation of bipotential gonads to either ovaries or testes in tilapia [44], [45], [46]. These results suggest that molecular sex determination of tilapia occurs at approximately 5 dah. Sex reversal could also be achieved by treated XX tilapia with androgens during this critical period [76]. However, the absence of both androgen and estrogen syntheses in early XY gonadal development has been reported in tilapia and rainbow trout [26], [27]. The transcriptomic data collected in the current work further confirmed the production of estrogen by XX gonads but not by XY gonads at 5 dah because all steroidogenic enzyme genes (including *cyp19a1a* and *cyp19a1b*) were up-regulated in the former while most of the steroidogenic enzyme genes were not expressed at all in the latter. *Cyp11b2* was undetectable in both XX and XY gonads at 5 dah, and there was no 11-keto-testosterone produced by undifferentiated XX or XY gonads. Consistent with these data, all estrogen and androgen receptors were expressed in the XX gonads, but only estrogen receptors and no androgen receptors were expressed in the XY gonads at 5 dah. Collectively, these data again suggest that endogenous estrogen is critical to ovarian determination in tilapia and that androgen is not necessary for testicular determination. During later stages (at 90 and 180 dah), the expression profiles of all steroidogenic enzymes except for *hsd3b2*, *hsd17b1*, and *cyp19a1a* were reversed. These three enzymes are involved in estrogen synthesis. Together with the fact that administration of the aromatase inhibitor fadrozole to three-month-old XX fish whose gonads had differentiated phase II and III oocytes resulted in sex reversal (unpublished data), we can conclude that estrogen is critical not only to sex determination but also to the maintenance of sex phenotype in tilapia.

For sex steroids to exert their effects, typically, their receptors must also be present. *Esr2a* and *esr2b* expression in XY gonads at 5 dah explains the susceptibility of XY fish to feminization by exogenous estrogen treatment even though endogenous steroidogenesis has not yet started by this stage. Correspondingly, the presence of androgen receptors (*ar1* and *ar2*) in XX gonads at 5 dah explains previous results detailing sex reversal upon exogenous androgen treatment. The enhanced expression of *esr2b* in XY gonads at all four developmental stages examined also confirms earlier findings and suggests that this nuclear receptor plays an important role in normal testicular differentiation and in XX female-to-male sex reversal [77].

## Supporting Information

Figure S1 **Scatter plots showing similar gene expression profiles in XX and XY tilapia gonads between 90 and 180 dah (FDR≤10^−2^ and |log_2_ (90 dah_RPKM/180 dah_RPKM)|≥1).** Gonads of the same sex displayed the highest similarity in gene expression pattern at these two developmental stages. “SEGs” indicate genes specifically expressed either at 90 or 180 dah. “DIGs” indicate genes differentially expressed between 90 and 180 dah. “COGs” indicate genes co-expressed at both 90 and 180 dah.
(TIF)

Figure S2 **Venn diagram of COGs across all four developmental stages of tilapia gonads.** Some 2,978 COGs were identified in both XX and XY gonads throughout all four stages of development.
(TIF)

Figure S3 **Venn diagram of XX-enhanced genes across all four developmental stages.** The 187 XX-enhanced genes included 11 genes that always exhibited higher levels of expression at four developmental stages, 12 genes that exhibited higher levels of expression at 5, 30, and 90 dah, 16 genes that exhibited higher levels of expression at 5, 30, and 180 dah, and 181 genes that exhibited higher levels of expression at 5, 90, and 180 dah.
(TIF)

Figure S4 **Venn diagram of XY-enhanced genes across all four developmental stages.** The 1,358 XY-enhanced genes included 225 genes that always exhibited higher levels of expression at four developmental stages, 323 genes that exhibited higher levels of expression at 5, 30, and 90 dah, 255 genes that exhibited higher levels of expression at 5, 30, and 180 dah, and 1,230 genes that exhibited higher levels of expression in 5, 90, and 180 dah.
(TIF)

Table S1 Full names and symbols of genes mentioned in the main text.
(DOC)

Table S2 Classification standards for genes expressed in tilapia gonads.
(DOC)

Table S3 Primers used for validation.
(DOC)

Table S4 Expression profiles of four characterized DIGs from RNA-seq data.
(DOC)

Table S5 List of the 2,978 COGs at all four developmental stages.
(XLSX)

Table S6 List of the 187 XX-enhanced genes. The 11 genes shown in yellow color were up-regulated in XX samples at all four stages of development. As stated in the materials and methods section, SEGs and DIGs expressed at 5 dah and in at least two other developmental stages in XX and XY gonads were considered XX- and XY-enhanced genes, respectively. Genes lacking FDR and |log_2_ (XX_RPKM/XY_RPKM)| at at least one stage were considered unable to meet the selection criteria at the stage in question but to meet these criteria for the other three stages.
(XLSX)

Table S7 List of the 1,358 XY-enhanced genes. 225 genes with yellow color were XY up-regulated genes at all four developmental stages. As stated in the materials and methods section, SEGs and DIGs expressed at 5 dah and in at least two other developmental stages in XX and XY gonads were considered to be XX- and XY-enhanced genes, respectively. Genes lacking FDR and |log_2_ (XX_RPKM/XY_RPKM)| at one stage were considered unable to meet the selection criteria for the stage in question but to meet these criteria for the other three stages.
(XLSX)
