# Supplementary figures and images for: Characterization of Gonadal Transcriptomes from Nile Tilapia (Oreochromis niloticus) Reveals Differentially Expressed Genes
Source: PLoS One. 2013 May 3;8(5):e63604. doi: 10.1371/journal.pone.0063604 (PMC3643912; doi:10.1371/journal.pone.0063604)

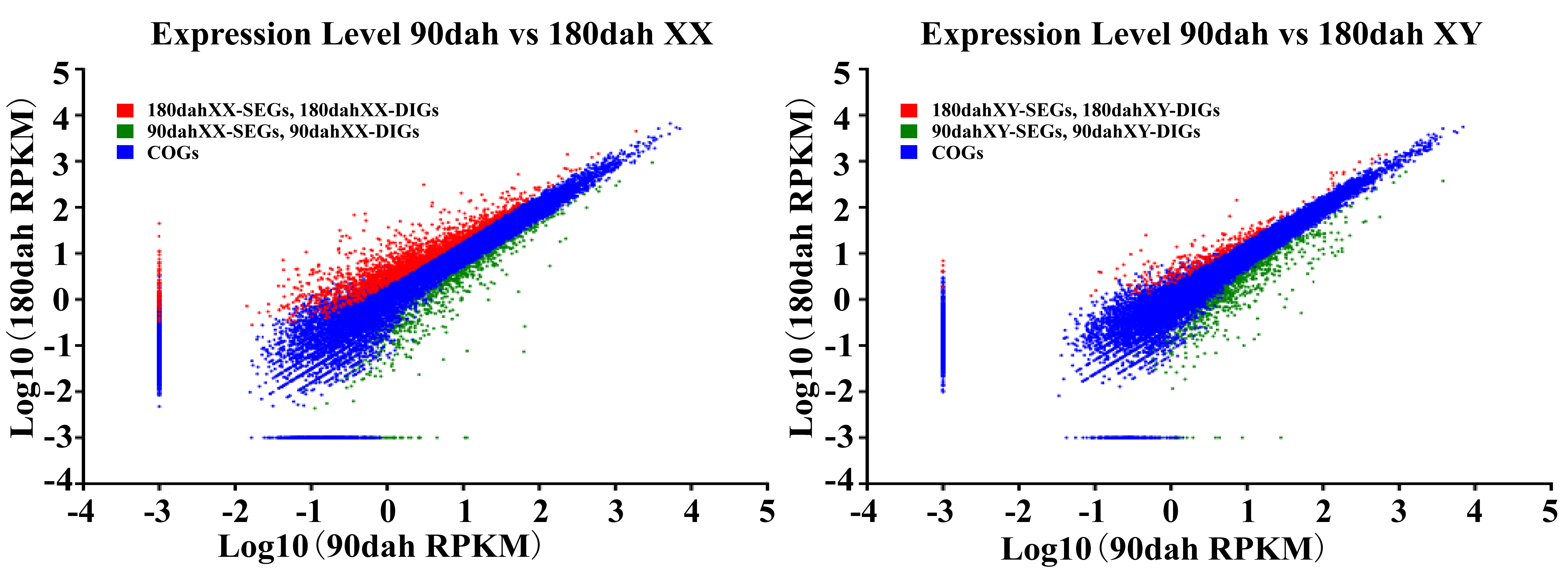

Supplement: Figure S1 — Scatter plots showing similar gene expression profiles in XX and XY tilapia gonads between 90 and 180 dah (FDR≤10−2 and |log2 (90 dah_RPKM/180 dah_RPKM)|≥1). Gonads of the same sex displayed the highest similarity in gene expression pattern at these two developmental stages. “SEGs” indicate genes specifically expressed either at 90 or 180 dah. “DIGs” indicate genes differentially expressed between 90 and 180 dah. “COGs” indicate genes co-expressed at both 90 and 180 dah. (TIF) [file pone.0063604.s001.tif]

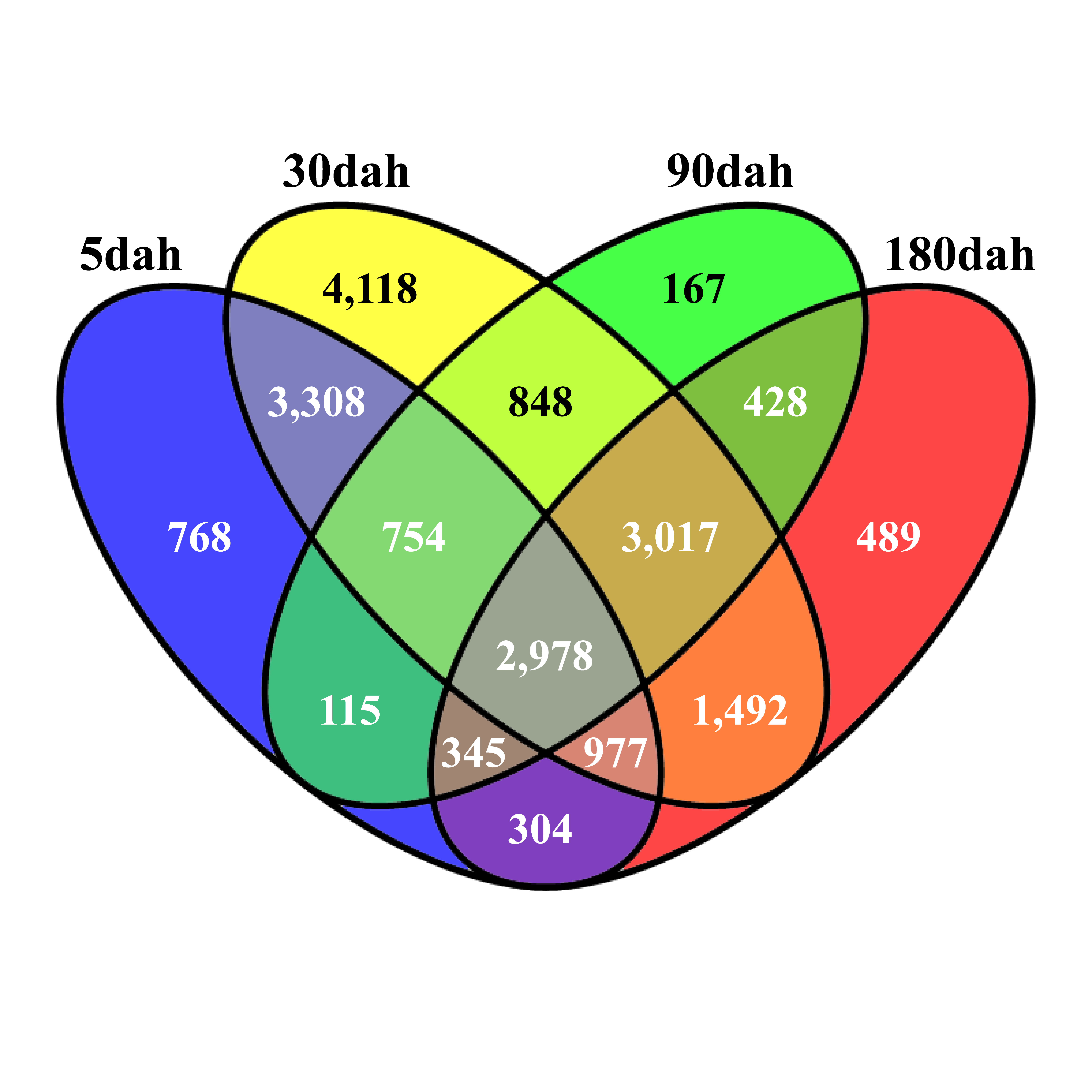

Supplement: Figure S2 — Venn diagram of COGs across all four developmental stages of tilapia gonads. Some 2,978 COGs were identified in both XX and XY gonads throughout all four stages of development. (TIF) [file pone.0063604.s002.tif]

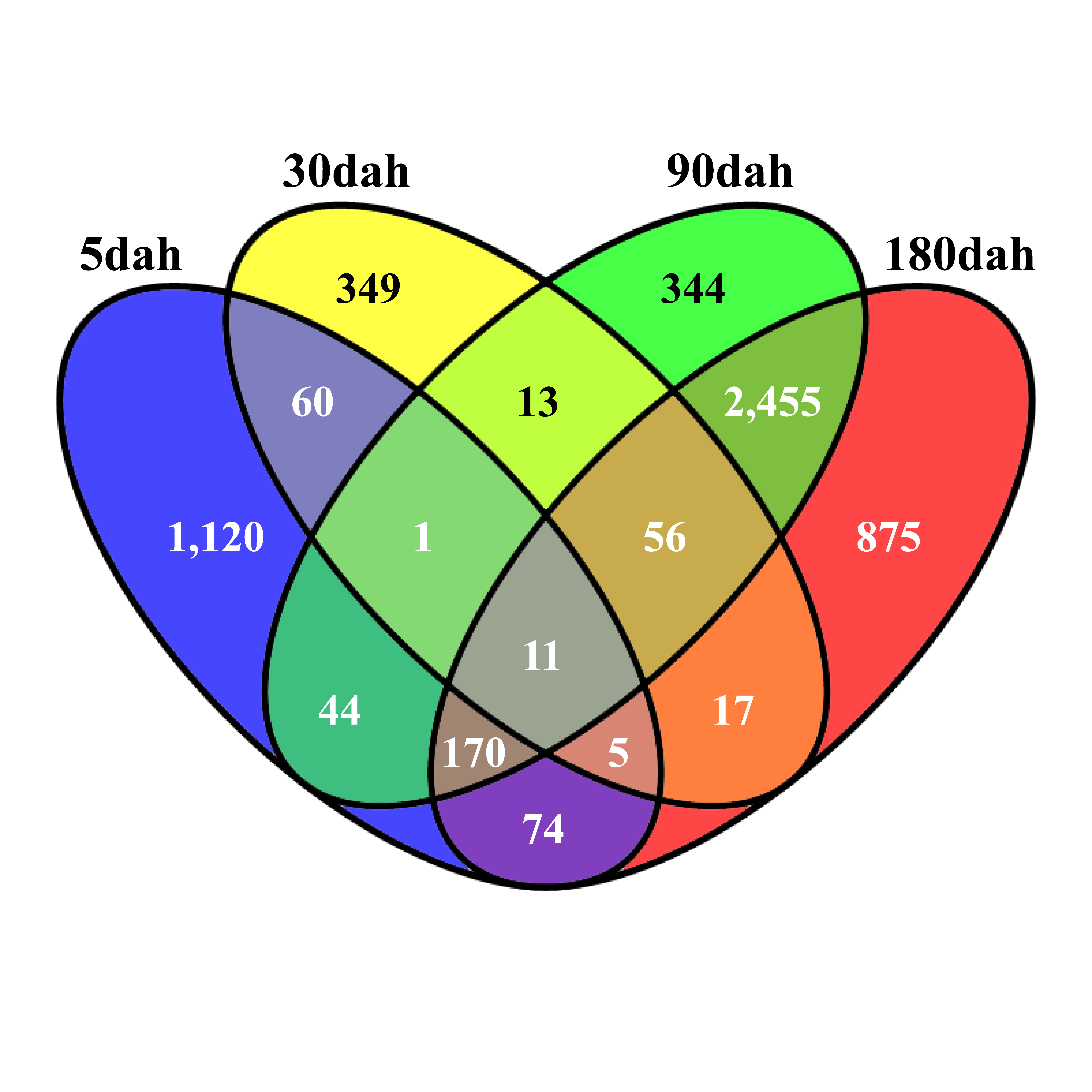

Supplement: Figure S3 — Venn diagram of XX-enhanced genes across all four developmental stages. The 187 XX-enhanced genes included 11 genes that always exhibited higher levels of expression at four developmental stages, 12 genes that exhibited higher levels of expression at 5, 30, and 90 dah, 16 genes that exhibited higher levels of expression at 5, 30, and 180 dah, and 181 genes that exhibited higher levels of expression at 5, 90, and 180 dah. (TIF) [file pone.0063604.s003.tif]

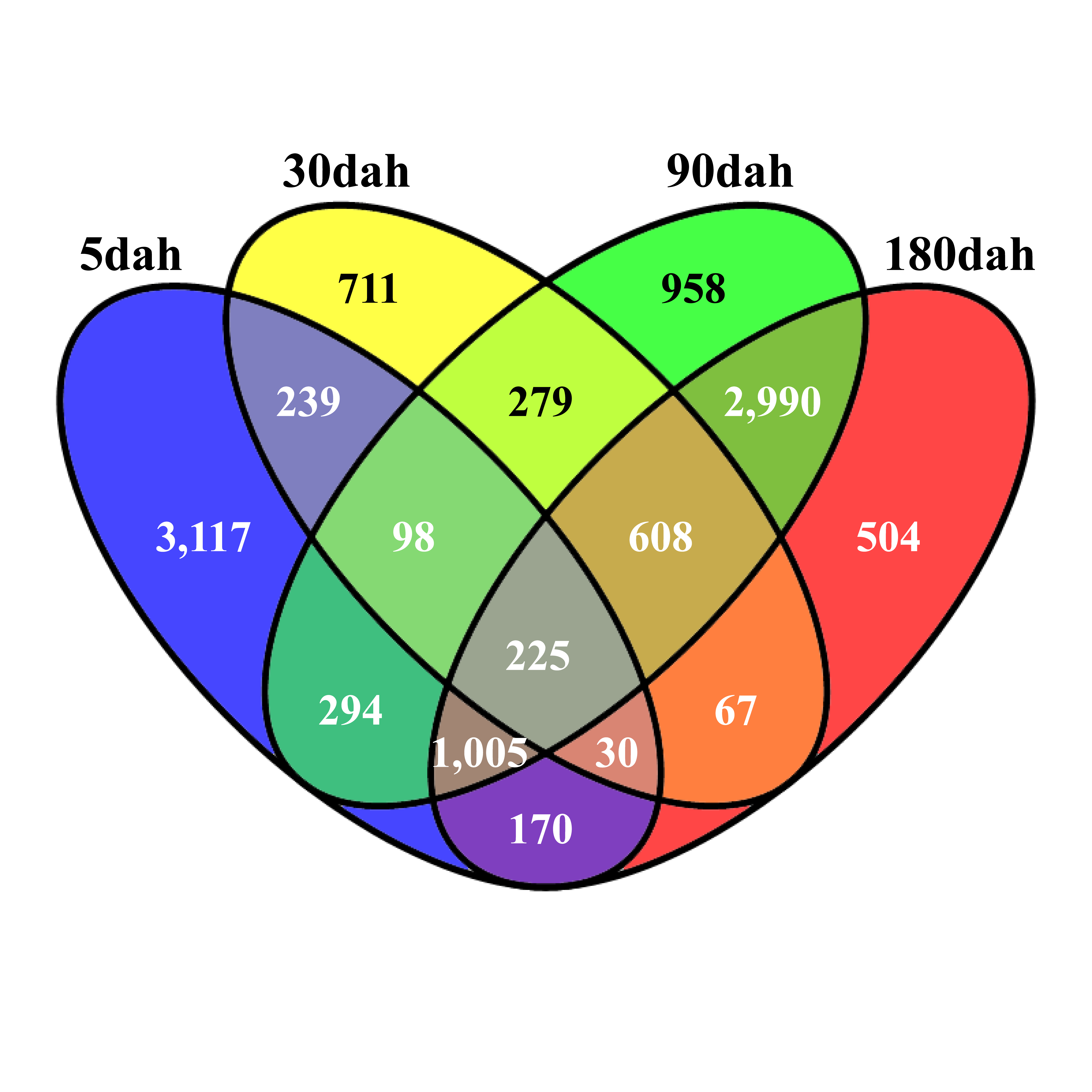

Supplement: Figure S4 — Venn diagram of XY-enhanced genes across all four developmental stages. The 1,358 XY-enhanced genes included 225 genes that always exhibited higher levels of expression at four developmental stages, 323 genes that exhibited higher levels of expression at 5, 30, and 90 dah, 255 genes that exhibited higher levels of expression at 5, 30, and 180 dah, and 1,230 genes that exhibited higher levels of expression in 5, 90, and 180 dah. (TIF) [file pone.0063604.s004.tif]
