# Supplementary material for: Characterization of Gonadal Transcriptomes from Nile Tilapia (Oreochromis niloticus) Reveals Differentially Expressed Genes
Source: PLoS One. 2013 May 3;8(5):e63604. doi: 10.1371/journal.pone.0063604 (PMC3643912; doi:10.1371/journal.pone.0063604)
Supplement: Table S1 — Full names and symbols of genes mentioned in the main text. (DOC) [file pone.0063604.s005.doc]

**Table S1. Full names and symbols of genes mentioned in the main text.**

| Gene symbols | GenBank ID | Gene full names |
| --- | --- | --- |
| *42sp50* | XM_003439246 | Elongation factor 1-alpha |
| *amhy* |  | a Y-linked anti-Müllerian hormone duplication |
| *ar1* | XM_003445816 | androgen receptor alpha |
| *ar2* | AB045212 | androgen receptor beta |
| *arhgap42* | XM_003446744 | Rho GTPase activating protein 42 |
| *cyp11a1* | XM_003440441 | cytochrome P450, family 11, subfamily A, polypeptide 1 |
| *cyp11b2* | FJ713103 | cytochrome P450, family 11, subfamily B, polypeptide 2 |
| *cyp17a1* | AB292401 | cytochrome P450, family 17, subfamily A, member 1 |
| *cyp17a2* | EF423917 | cytochrome P450, family 17, subfamily A, member 2 |
| *cyp19a1a* | U72071 | cytochrome P450, family 19, subfamily A, polypeptide 1a. |
| *cyp19a1b* | AF295761 | cytochrome P450, family 19, subfamily A, polypeptide 1b |
| *dax1* | AY135397 | dosage-sensitive sex reversal, adrenal hypoplasia critical region, on chromosome X, gene 1 |
| *dazap1* | XM_003449023 | DAZ-associated protein 1 and autoantigen |
| *ddx46* | XM_003451406 | DEAD (Asp-Glu-Ala-Asp)–box family of RNA helicases 46 |
| *Dmrt1* | AF203489 | doublesex and mab-3 related transcription factor 1 |
| *Dmw* |  | DM domain gene on the W chromosome |
| *dmy* |  | DM domain gene on the Y chromosome |
| *eef1a1b* | XM_003454630 | eukaryotic translation elongation factor 1 alpha 1 |
| *esr1* | U75604 | estrogen receptor alpha |
| *esr2a* | U75605 | estrogen receptor beta 1 |
| *esr2b* | DQ462608 | estrogen receptor beta 2 |
| *fgf16* | JN860432 | Fibroblast growth factor-16 |
| *fgf20b* | JN860434 | Fibroblast growth factor-20b |
| *foxh1* | XM_003443494 | forkhead box H1 |
| *foxj1a* | XM_003442567 | forkhead box j1a |
| *foxl2* | AY554172 | forkhead transcription factor 1 |
| *gpc4* |  | *glypican 4* |
| *hsd11b2* | AY190043 | 11β-Hydroxysteroid dehydrogenase type 2 |
| *hsd3b1* | EU827279 | Type I 3β-hydroxysteroid dehydrogenase/Δ5-Δ4-isomerase |
| *hsd3b2* | EU827281 | Type II 3β-hydroxysteroid dehydrogenase/Δ5-Δ4-isomerase |
| *hsd17b1* | AY663853 | 17β-Hydroxysteroid dehydrogenase type 1 |
| *hsd17b8* | AY663855 | 17β-Hydroxysteroid dehydrogenase type 8 |
| *hsd17b12a1* | XM_003455397 | 17β-Hydroxysteroid dehydrogenase type 12a1 |
| *hsd17b12a2* | XM_003450799 | 17β-Hydroxysteroid dehydrogenase type 12a2 |
| *hsd17b14* | XM_003442518 | 17β-Hydroxysteroid dehydrogenase type 14 |
| *hsp70* |  | heat-shock protein 70 |
| *igfbp3* |  | Insulin-like growth factor-binding protein 3 |
| *irf9y* |  | interferon regulatory factor 9 |
| *lamc2* | XM_003458940 | laminin, gamma 2 |
| *m33* |  | a mouse homologue of Drosophila Polycomb gene |
| *nanos2* | XM_003439316 | nanos homolog 2 |
| *par-3* |  | par-3 partitioning defective 3 homolog B |
| *pen* | AF176651 | pendulin |
| *rpl12* | XM_003437601 | ribosomal protein L12 |
| *rpl3* | XM_003442604 | ribosomal protein L3 |
| *rpl5* | XM_003437785 | ribosomal protein L5 |
| *sf1* | AB060814 | steroidogenic factor 1 |
| *snip1* | XM_003450516 | Smad nuclear interacting protein 1 |
| *sox9* | XM_003450119 | SRY-related high mobility group-box gene9 |
| *Sry* |  | sex-determining region Y |
| *star1* | XM_003445605 | Steroidogenic acute regulatory protein 1 |
| *star2* | XM_003441906 | Steroidogenic acute regulatory protein 2 |
| *wnt5a* | XM_003448288 | wingless-type MMTV integration site family, member 5A |
