# Supplementary material for: Characterization of Gonadal Transcriptomes from Nile Tilapia (Oreochromis niloticus) Reveals Differentially Expressed Genes
Source: PLoS One. 2013 May 3;8(5):e63604. doi: 10.1371/journal.pone.0063604 (PMC3643912; doi:10.1371/journal.pone.0063604)
Supplement: Table S2 — Classification standards for genes expressed in tilapia gonads. (DOC) [file pone.0063604.s006.doc]

**Table S2.** Classification standards for genes expressed in tilapia gonads.

| Full name | Abbreviation | identification standards | | |
| --- | --- | --- | --- | --- |
|  |  | FDR and log_2_ (XX_RPKM/XY_RPKM) | Gonad |  |
| XX specifically expressed gene | XX-SEG | FDR≤10^-2^ and log_2_ (XX_RPKM/XY_RPKM)≥1 | XX |  |
| XY specifically expressed gene | XY-SEG | FDR≤10^-2^ and log_2_ (XX_RPKM/XY_RPKM)≤-1 | XY |  |
| specifically expressed gene that did not meet statistical criteria | ND-SEG | FDR>10^-2^ or -1<log_2_ (XX_RPKM/XY_RPKM)<1 | XX or XY |  |
| XX differentially expressed gene | XX-DIG | FDR≤10^-2^ and log_2_ (XX_RPKM/XY_RPKM)≥1 | XX and XY |  |
| XY differentially expressed gene | XY-DIG | FDR≤10^-2^ and log_2_ (XX_RPKM/XY_RPKM)≤-1 | XX and XY |  |
| XX and XY co-expressed gene | COG | FDR >10^-2^ or -1<log_2_ (XX_RPKM/XY_RPKM)<1 | XX and XY |  |
