# Supplementary material for: Characterization of Gonadal Transcriptomes from Nile Tilapia (Oreochromis niloticus) Reveals Differentially Expressed Genes
Source: PLoS One. 2013 May 3;8(5):e63604. doi: 10.1371/journal.pone.0063604 (PMC3643912; doi:10.1371/journal.pone.0063604)
Supplement: Table S3 — Primers used for validation. (DOC) [file pone.0063604.s007.doc]

**Table S3. Primers used in the present study.**

| **Primers** | **Sequences** |
| --- | --- |
| ***β-actin*-F** | 5'-GGCATCACACCTTCTACAACGA-3'* |
| ***β-actin*-R** | 5'-ACGCTCTGTCAGGATCTTCA-3'* |
| ***42sp50*-F** | 5'-CCTGGGTGCTAGACAAGATGAA-3' |
| ***42sp50*-R** | 5'-GTCAGCCTGTGATGTCCCAGTTAT-3' |
| ***eef1a1b*-F** | 5'-GGCTGCTGCCCCAAACGCTCAC-3' |
| ***eef1a1b*-R** | 5'-AGGCAGAGAACTGTAACGCAAACC-3' |
| ***foxh1*-F** | 5'-CGTTAGCTGAGATCCTCAAGGAGAT-3' |
| ***foxh1*-R** | 5'-CTGTGGCTTACCTGGGTCTTTTAAT-3' |
| ***foxj1a*-F** | 5'-CCCACCTGGCAGAACTCGAT-3' |
| ***foxj1a*-R** | 5'-GGGCGGCATTCGTCTCTT-3' |

Note: * indicate primers for *β-actin* were according to Yoshiura *et al.* [55].
