# Supplementary material for: Characterization of Gonadal Transcriptomes from Nile Tilapia (Oreochromis niloticus) Reveals Differentially Expressed Genes
Source: PLoS One. 2013 May 3;8(5):e63604. doi: 10.1371/journal.pone.0063604 (PMC3643912; doi:10.1371/journal.pone.0063604)
Supplement: Table S4 — Expression profiles of four characterized DIGs from RNA-seq data. (DOC) [file pone.0063604.s008.doc]

**Table S4.** Expression profiles of four characterized DIGs from RNA-seq data.

| **Gene** | **5dah XX** | **5dah XY** | **30dah XX** | **30dah XY** | **90dah XX** | **90dah XY** | **180dah XX** | **180dah XY** |
| --- | --- | --- | --- | --- | --- | --- | --- | --- |
| ***foxl2*** | 328.37 | 104.06 | 17.06 | 1.39 | 13.58 | 0.32 | 26.46 | 0.00 |
| ***fgf16*** | 0.00 | 1.81 | 0.36 | 1.16 | 26.90 | 2.53 | 12.99 | 0.84 |
| ***fgf20b*** | 0.10 | 0.46 | 2.92 | 4.43 | 33.65 | 2.80 | 38.09 | 1.36 |
| ***dmrt1*** | 29.16 | 555.17 | 16.60 | 155.12 | 4.50 | 244.73 | 4.54 | 282.56 |

Note: RNA-seq data are shown as RPKM, which are consistent with the published data [46,57].
